# Isolation, Molecular Characterization and Probiotic Potential of Lactic Acid Bacteria in Saudi Raw and Fermented Milk

**DOI:** 10.1155/2018/7970463

**Published:** 2018-07-25

**Authors:** Maged S. Bin Masalam, Ahmed Bahieldin, Mona G. Alharbi, Saad Al-Masaudi, Soad K. Al-Jaouni, Steve M. Harakeh, Rashad R. Al-Hindi

**Affiliations:** ^1^Department of Biology, Faculty of Science, King Abdulaziz University, Jeddah, Saudi Arabia; ^2^Department of Pediatric Hematology/Oncology, Yousef Abdullatif Jameel Chair of Prophetic Medicine Application, King Abdulaziz University Hospital, Faculty of Medicine, KAU, Saudi Arabia; ^3^Special Infectious Agents Unit, King Fahd Medical Research Center, Yousef Abdullatif Jameel Chair of Prophetic Medicine Application, KAU, Saudi Arabia

## Abstract

Probiotic bacteria can confer health benefits to the human gastrointestinal tract. Lactic acid bacteria (LAB) are candidate probiotic bacteria that are widely distributed in nature and can be used in the food industry. The objective of this study is to isolate and characterize LAB present in raw and fermented milk in Saudi Arabia. Ninety-three suspected LAB were isolated from thirteen different types of raw and fermented milk from indigenous animals in Saudi Arabia. The identification of forty-six selected LAB strains and their genetic relatedness was performed based on 16S rDNA gene sequence comparisons. None of the strains exhibited resistance to clinically relevant antibiotics or had any undesirable hemolytic activity, but they differed in their other probiotic characteristics, that is, tolerance to acidic pH, resistance to bile, and antibacterial activity. In conclusion, the isolates* Lactobacillus casei* MSJ1,* Lactobacillus casei* Dwan5,* Lactobacillus plantarum* EyLan2, and* Enterococcus faecium* Gail-BawZir8 are most likely the best with probiotic potentials. We speculate that studying the synergistic effects of bacterial combinations might result in a more effective probiotic potential. We suspect that raw and fermented milk products from animals in Saudi Arabia, especially Laban made from camel milk, are rich in LAB and have promising probiotic potential.

## 1. Introduction

Probiotic bacteria can confer health benefits to the human gastrointestinal tract [1–3]. Lactic acid bacteria (LAB) are candidate probiotic bacteria [4] that are widely distributed in nature and can be used in the food industry [5]. LAB are a group of microaerophilic or anaerobic Gram-positive bacteria that are unable to form spores or produce catalase and are characterized by the absence of the cytochrome system [6, 7] and the ability to produce antimicrobials for biopreservation [8, 9]. Certain foods, including dairy products, for example, yogurt, are considered good sources of probiotics [10]. The majority of microbiota in raw milk and fermented milk products include the genera* Lactobacillus*,* Enterococcus*,* Lactococcus*,* Leuconostoc*,* Pediococcus*,* Oenococcus*,* Carnobacterium*,* Streptococcus*, and* Weissella* [11–13]. The most obvious benefits of LAB fermentation include increased food palatability and improved shelf life [14]. LAB are generally recognized as safe (GRAS) because they are able to produce bacteriocins and their consumption confers several health benefits, such as controlling intestinal infections, improving lactose utilization, lowering blood ammonia levels, providing efficient resistance against gastric acid and bile [11, 15–19], influencing the immune system, and lowering serum cholesterol levels [20, 21]. LAB also adhere to the gastrointestinal tract and confer pathogen inhibition [11, 22, 23]. Interestingly, the presence of LAB resulted in no change or small changes in the abundance of other intestinal microbial groups [24].

DNA-based molecular identification of the 16S rRNA gene can discriminate between closely related bacterial species [25–29]. Amplified ribosomal DNA restriction analysis (ARDRA) and randomly amplified polymorphic DNA (RAPD) were successfully used in discriminating LAB [30, 31]. The approaches include PCR-RFLP followed by direct sequencing of the 16S rDNA gene and a BLAST search against the sequences of other organisms that are available at the National Center for Biotechnology Information (NCBI) [32].

The isolation, identification, and characterization of novel LAB strains have two benefits. The first is to reveal the characteristic taxonomy of the LAB and the second is to obtain promising beneficial and functional probiotic LAB [33, 34]. There is little research regarding the isolation and characterization of LAB from dairy products. Therefore, the aim of the present work was to isolate and to identify, at the molecular level, the lactic acid bacteria contained in raw milk and fermented milk which were produced indigenously in Saudi Arabia. These LAB were evaluated for their functional traits, probiotic properties, and ability to inhibit the growth of pathogenic and food poisoning bacteria.

## 2. Materials and Methods

### 2.1. Samples and Media

Thirteen (0.5 kg) samples of raw milk and traditional fermented milk from indigenous animals procured in Jeddah Province, Saudi Arabia, were used in this study. These samples were collected from the local market and were stored in a fridge until use. MRS agar and MRS broth media (Oxoid^TM^, Thermo Fisher Scientific, USA) were used to isolate and support the growth of LAB and to inhibit the growth of unwanted bacteria [35]. MRS agar and broth were also used to enumerate the LAB. M17 agar and M17 broth (Oxoid, Thermo Fisher Scientific, USA) were used for isolating the streptococci in dairy products [36, 37]. Blood agar (Oxoid, Thermo Fisher Scientific, USA) and Muller-Hinton agar media (MHA) (HiMedia, India) were used to evaluate hemolytic activity and antimicrobial activity of the LAB, respectively.

### 2.2. Isolation of LAB and Maintenance Method

50 g of the samples was placed into sterile stomacher bags and then were diluted (1:10) with MRS broth, processed to enrich the LAB by stomaching, sealed, and incubated overnight at 30°C [38]. Lactic acid bacteria were isolated by 5-fold serial dilutions. At the beginning, 1 mL of sample stomachate was added to 9 mL of sterile physiological water (0.85% NaCl) and was further serially diluted. Then, 0.1 mL aliquots of the samples' suitable dilutions were plated onto MRS agar and M17 agar. The plates were incubated at 37°C for 24-48 h in anaerobic jars using the AnaeroGen™ anaerobic system (Oxoid, Thermo Fisher Scientific, USA) [25, 39, 40] and in microaerobic conditions (5% CO_2_) [41]. MRS agar plates were used to enumerate the initial growth of the LAB in each sample [25]. All experiments were performed in triplicate. The resulting isolates were randomly selected from the medium surface and were streaked twice on fresh MRS to be purified [35]. The isolated strains were maintained by culturing them in MRS broth medium at 30°C and then storing at −80°C in MRS broth containing 50% (v/v) glycerol. MRS broth and M17 broth were used in all subsequent experiments [42, 43]. Isolates from the stocks were subcultured in MRS broth for further studies.

### 2.3. Phenotypic and Genotypic Identifications

For all of the isolated strains, the colony morphology on MRS solid medium and M17 agar was determined visually and their motility was recorded using the hanging drop technique. Gram staining was performed to determine the cell morphology and Gram stain reaction of the isolates. Catalase and cytochrome oxidase tests were also performed [39, 42]. As for genotypic identification, cell pellets were harvested from 2 mL of overnight cultures (up to 2 × 10^9^ bacterial cells) of LAB grown in MRS broth by centrifugation for 10 min at 5,000 ×g, and the supernatant was discarded. Then, DNA extraction was done using a GeneJET Genomic DNA Purification Kit (cat. no. K0721, Thermo Fisher Scientific, Waltham, MA, USA) following the manufacturer's instructions. PCR was carried out and the universal primers were designed and synthesized (Medicbiotrade, Germany) to amplify nearly the entire region of the 16S rDNA gene; the primers were 16S:F27 (forward: 5′-AGAGTTTGATCCTGGCTCAG-3′) and 16S:R27 (reverse: 5′-AAGGAGGTGATCCAGCCGCA-3′). The primers were designed to amplify the 16S rDNA gene (~1.6 kb, [44]) with GC contents of 50-60% for a higher melting temperature (Tm) to avoid nonspecific amplification [39]. PCR conditions were one cycle of 5 min of initial denaturation at 95°C, followed by 35 cycles of amplification. Each cycle consisted of denaturation at 95°C for 30 sec, annealing at 58°C for 30 sec, and extension at 72°C for 1 min. The last cycle was 10 min at 72°C as a postextension step. Electrophoresis in 0.5x TBE buffer was conducted on an 0.8% agarose gel for the amplicons with a 1 kb DNA standard (TrackIt, Invitrogen, Waltham, MA, USA), and the gels were stained with ethidium bromide and photographed. The amplicons were purified, they were sequenced for the 16S gene at Macrogen Inc. (Seoul, Korea), and they were BLAST-searched to detect similar sequences in the NCBI database (https://www.ncbi.nlm.nih.gov) using the Discovery Studio Gene v1.5 (DSGene v1.5) program [32, 42]. A fragment of ~1000 nt involving five variable (V) regions was further utilized. Binary data metrics were entered into TFPGA (version 1.3) and were analyzed using a qualitative route to generate a similarity coefficient. The dissimilarity coefficients were used to construct dendrograms by the sequential hierarchical and nested clustering (neighbor joining (NJ)) method with NTSYSpc (version 2.10, Exeter Software).

### 2.4. Antibiotic Susceptibility, Hemolytic Activity, and Acid Bile Tolerance

The antibiotic susceptibility of the identified LAB was determined by using the agar disk diffusion method [45]. Fifteen antibiotics (Oxoid, Thermo Fisher Scientific, USA) were selected as representatives of the different classes of clinically important antibiotics. Inoculates (10^6^ CFU mL^−1^) of the LAB were swabbed onto the surfaces of Muller-Hinton agar plates and, then, the disks of antibiotics (amoxicillin/25 *μ*g, penicillin G/10 units, ceftriaxone/30 *μ*g, cefoxitin/30 *μ*g, tobramycin/10 *μ*g, oxacillin/1 *μ*g, bacitracin/10 units, chloramphenicol/30 *μ*g, polymyxin B/300 units, gentamycin/10 *μ*g, neomycin/30 *μ*g, clindamycin/2 *μ*g, vancomycin/256 *μ*g, nitrofurantoin/300 *μ*g, and nalidixic acid/30 *μ*g) were applied onto the surface of the plates and incubated at 37°C and 5% CO_2_ for 24 h [17, 46]. The plates were examined for the presence of inhibition zones around the antibiotic disks [43]. The inhibitory effect of the antibiotics was expressed as the diameter (mm) of the inhibition zones.

Bacterial cells were grown on Columbia blood agar (Oxoid, Thermo Fisher Scientific, USA), supplemented with 5% (v/v) human blood (obtained from King Abdulaziz University Hospital, Jeddah, KSA) to determine their ability to produce different types of hemolysins. Plates were incubated at 37°C in anaerobic jars (Oxoid, Thermo Fisher Scientific, USA) with gas-generating kits. After 24-hour and 72-hour incubation periods, the results were recorded. A clear zone on the blood agar plates was considered a positive result [42].

Tolerance of the LAB to acid and bile was determined as described [47]. All strains were grown at 37°C for 24 h in MRS broth and were suspended to an approximate cell concentration of 10^8^ CFU mL^−1^ (using turbidity standard McFarland 0.5) in MRS broth adjusted to pH 3.0 for 2 h, and the cells were then incubated in 0.5% w/v bile (Sigma-Aldrich, France) for 4 h. These conditions were chosen to represent the time required for the bacteria to pass through the gastrointestinal system and the pH value and bile concentration found in the stomach and intestine, respectively. Bacterial viability was assessed by enumeration on MRS agar plates at zero time and at the end of incubation.

### 2.5. Evaluation of Antibacterial Activity of Identified Strains

The agar well diffusion method was used to adequately investigate the antibacterial potential of the 46 identified LAB as described by Schillinger et al. (1996), Chahad et al. (2012), and Messaoudi et al. (2012a). Suspensions of seven indicator bacterial cultures, namely,* Staphylococcus aureus* ATCC 25923, methicillin-resistant* Staphylococcus aureus* (MRSA) ATCC 43330,* Escherichia coli *ATCC 25922,* Salmonella* spp.,* Shigella sonnei* ATCC 25931,* Enterococcus faecalis* ATCC 29212, and* Listeria monocytogenes* ATCC 13932, provided by the Microbiology Laboratory of King Abdulaziz University Hospital, Jeddah, KSA, were prepared using turbidity standard McFarland 0.5. Then, 150 *μ*L of these suspensions was inoculated onto MHA medium by the streaking plate method, and four wells were punched in each inoculated agar medium plate using a sterile cork borer (6 mm diameter). Cell-free supernatant (CFS) of LAB grown in MRS was prepared by centrifugation at 10,000 ×g for 20 min and 4°C. Then, 100 *μ*L aliquots of the CFS of each strain were pipetted into their designated wells on the plates. Finally, the susceptibility of the test organisms against MRS broth was taken as a control. All inoculated plates were incubated at 37°C for 18-24 h. After incubation, the diameter of each formed inhibition zone was measured twice using ruler; then, the average was taken to represent the antibacterial activity.

### 2.6. Statistical Analysis

Data were expressed as the mean ± standard deviation (SD) calculated over independent experiments performed in triplicate. For inference statistics, one-way analysis of variance (ANOVA) was applied, and it was performed with the Statistical Package for the Social Sciences (SPSS/PC, version 20.0).

## 3. Results

### 3.1. Isolation and Characterization of LAB

A total of 93 lactic acid bacteria (LAB) were isolated from the 13 collected samples of locally produced raw milk and fermented milk, such as fresh raw milk, frozen raw milk, fresh cheese, salty cheese, cooked cheese, stirred yogurt (Laban), qeshta (cream), madheer (dried fermented milk), yogurt, and butter. These samples originated from different animal sources, including cows, goats, and camels. The LAB counts under microaerobic incubation conditions were higher than those under anaerobic conditions. For example, the mean counts (per gram of sample) in the samples of qeshta made of cow milk and stirred yogurt “Laban” made of camel milk grown for 48 h under microaerobic incubation conditions were between 4.1 × 10^6^ ± 0.2 × 10^6^ and 2.2 × 10^10^ ± 0.2 × 10^10^ CFU g^−1^, respectively, while these counts were between 4.7 × 10^2^ ± 0.6 × 10^2^ and 2.7 × 10^10^ ± 0.3 × 10^9^ CFU g^−1^, respectively, under anaerobic incubation conditions.

The colony morphologies of the isolates were visually observed on the surface of MRS solid medium; the color varied from white to pale creamy, the shape was circular, and the size ranged from 0.5 to 4 mm in diameter. Most strains (92.47%) were Gram-positive. The isolated strains differed in their catalase and cytochrome oxidase activity, where 71% were negative for catalase activity and 72% were negative for cytochrome oxidase production activity. The motility of the isolated strains also differed when tested by the hanging drop technique, with 96% of the isolates being nonmotile.

**Figure 1:**
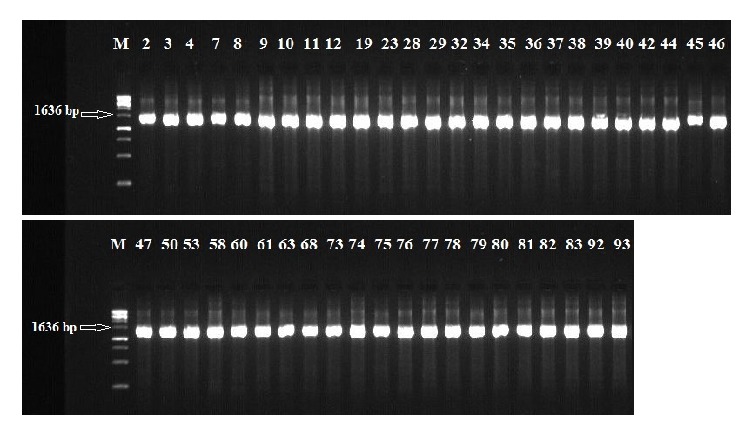
PCR amplification of the 16S rDNA from the isolated LAB on 0.8% agarose gel. M: 1 kb DNA marker (TrackIt, Invitrogen). Numbers 2-4, 7-12, 19, 23, 28, 29, 32, 34-40, 42, 44-47, 50, 53, 58, 60, 61, 63, 68, 73-83, 92, and 93 refer to the strains numbers in Table 1.

**Figure 2:**
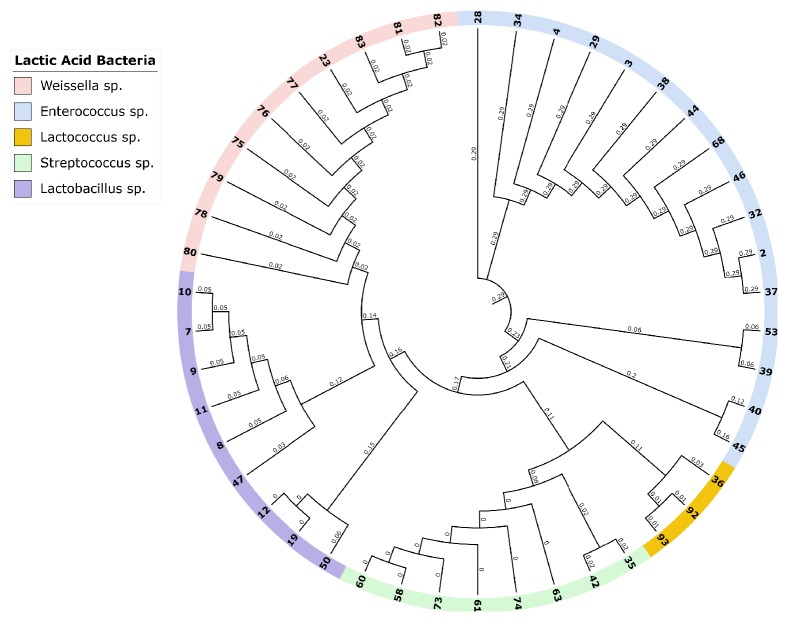
Phylogenetic neighbor joining (NJ) tree based on the 16S rDNA full-length sequences (~1.6 kb) of the 46 selected LAB strains of raw milk and fermented milk samples based on the results of sequence alignment. The numbers on the tree refer to the LAB strains in Table 1.

The 16S rDNA gene (~1.6 kb) was amplified from all of the isolated strains (Figure 1). Then, the amplicons were column-purified and sequenced. The resultant nucleotide sequences were BLAST-searched for homology with known sequences in the NCBI database. The results in Table 1 indicated that the nucleotide sequences of 46 strains aligned with the 16S rDNA sequences of 14 different species belonging to five genera, namely,* Enterococcus*,* Lactococcus*,* Lactobacillus*,* Streptococcus*, and* Weissella* (Figure 2). These strains showed identities of 99% (12 strains), 98% (14 strains), 97% (12 strains), 96% (6 strains), and 95% (2 strains). Analysis of the 16 strains aligned with the genus* Enterococcus *indicated that 12 strains (i.e., Rashad3, SMBM3, ZiNb3, Gail-BawZir8, NSJ2, Marwh2, Mona3, SSJ3, Adeb3, ESJ4, BagHom4, and Ma7Fod) might belong to the species* E. faecium,* while two strains (i.e., Etimad1 and She7R), one strain (i.e., Jeddah9), and one strain (i.e., Shbam40) might belong to the species* E. durans*,* L. lactis*, and* E. faecalis*, respectively. In studying the three strains of the genus* Lactococcus,* the results indicated that one strain (i.e., HadRami9) might belong to the species* L. lactis*, while two strains (i.e., Emad4 and ZSJ5) might belong to the species* L. garvieae*. Analysis of the nine strains of the genus* Lactobacillus* indicated that two strains (i.e., Hadhramaut4 and Musallam2), four strains (i.e., MSJ1, BgShn3, MasaLam7, and Dwan5), one strain (i.e., NMBM1), one strain (i.e., EyLan2), and one strain (i.e., EMBM2) might belong to the species* L. acidophilus*,* L. casei*,* L. paracasei*,* L. plantarum*, and* L. futsaii*, respectively. Two out of the eight strains of the genus* Streptococcus* (i.e., BinSlman8 and MaNaL33) might belong to the species* S. thermophilus*, while the rest (i.e., Omer9, Anwr4, Zaki1, Salam7, JmaL3, and Foad7) might belong to the species* S. equinus*. In addition, all ten strains of the genus* Weissella* (i.e., SaEd-7, AhMd8, Tarim4, NooR1, SaYun2, MuKalla5, SYary1, Sho7ir, Farag8, and A7Gaf) might belong to the species* W. confusa*.

### 3.2. Susceptibility of LAB to Antibiotics and Hemolytic Activity

The tested 46 LAB underwent an antibiotic susceptibility testing, and their growth was inhibited, to some extent, by the majority of the 15 tested antibiotics (Table 2). Interestingly, three antibiotics (bacitracin, gentamicin, and neomycin) had a great (~100%) inhibition effect against all the tested strains with a spectrum of inhibition zones of 14.0 ± 0.0 - 28.5 ± 0.71, 9.5 ± 0.71 - 40.0 ± 0.0, and 9.5 ± 0.71 - 31.5 ± 0.71 mm, respectively. On the other hand, penicillin G, tobramycin, and vancomycin inhibited the growth with a spectrum of inhibition zones of 15.0 ± 1.41 - 40.0 ± 1.41, 11.0 ± 1.41 - 23.5 ± 0.71, and 9.5 ± 0.71 - 23.5 ± 0.71 mm at lower levels of 89%, 73.9, and 67.4% inhibition effect on the tested strains, respectively. There were only four antibiotics (i.e., nalidixic acid, polymyxin B, oxacillin, and cefoxitin) that had low inhibition action at levels of 17.4%, 30.4%, 34.8%, and 45.7%, respectively. The actions of the antibiotics oxacillin, cefoxitin, and nalidixic acid were resisted by many strains of lactobacilli and* W. confusa*. Five strains (namely,* S. thermophilus* BinSlman8,* S. thermophilus* MaNaL33,* S. equinus* Omer9,* S. equinus* JmaL3, and* S. equinus* Foad7) were resistant to penicillin G, while 15 strains (32.6%) were resistant to vancomycin. These strains belong to* L. casei* (4 strains),* L. paracasei *NMBM1,* L. plantarum *EyLan2,* L. futsaii *EMBM2, and* W. confusa* (8 strains).

In our study, 32 (69.57%) of the tested strains were nonhemolytic (*γ*-hemolysis), while the remaining 14 (30.43%) strains exhibited *α*-hemolytic activity, and these were* S. thermophilus* BinSlman8,* S. thermophilus* MaNaL33,* W. confusa* SaEd-7,* W. confusa* AhMd8,* W. confusa* Tarim4,* W. confusa* NooR1,* W. confusa* SaYun2,* W. confusa* MuKalla5,* W. confusa* Sho7ir,* W. confusa* Farag8,* W. confusa* A7Gaf,* W. confusa* SYary1,* L. garvieae* ZSJ5, and* L. garvieae* Emad4 (Table 2).

### 3.3. Acid and Bile Tolerance

**Figure 3:**
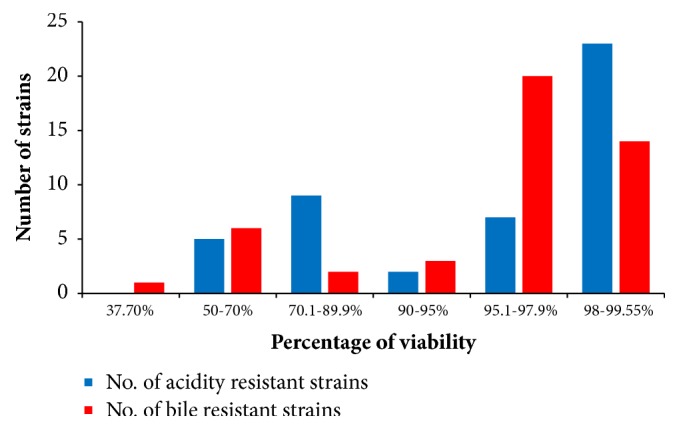
The percentage viability of the 46 LAB grouped according to their acidity and bile tolerance.

The results showed that the 46 tested strains tolerated the acidic condition by variable ratios. The percentage viability of 50-70%, 70.1-89.9%, 90-95%, 95.1-97.9%, and 98-99.55% occurred among 5, 9, 2, 7, and 23 strains, respectively (Table 2 and Figure 3). The most acid-tolerant strains were enterococci, such as* E. faecium* SMBM3,BagHom4, ZiNb3, Gail-BawZir8, ESJ4, NSJ2, Marwh2, SSJ3, Etimad1, and other LAB genera, such as* W. confusa* SaEd-7, AhMd8, Tarim4, NooR1, SaYun2, SYary1,* L. casei* MSJ1, BgShn3, Dwan5,* L. futsaii* EMBM2, and* L. lactis* HadRami9 (Table 2). The 46 LAB with acidity tolerances ranging from 52.85% to 99.55% were further tested for 4 h for 0.5% w/v bile tolerance. The obtained results showed that the survival percentages were 37.7%, 50-70%, 70.1-89.9%, 90-95%, 95.1-97.9%, and 98-99.55% for 1, 6, 2, 3, 20, and 14 strains, respectively (Table 2 and Figure 3). A total of 71.7% of the strains were bile-tolerant without any significant loss of viability (>95% survival). The most bile-tolerant strains were among enterococci, such as* E. faecium* ZiNb3,* E. faecium* Rashad3, and* E. faecium* SMBM3, and other species, such as* L. casei* BgShn3,* L. casei* Dwan5,* L. casei* MSJ1,* L. plantarum* EyLan2,* L. acidophilus* Musallam2,* L. paracasei* NMBM1,* S. equinus* Salam7,* L. garvieae* Emad4,* L. garvieae* ZSJ5, and* W. confusa* SYary1 (Table 2).

### 3.4. Antibacterial Activity of Isolated LAB

Spectra of the antibacterial activity of the CFS preparations for the 46 LAB against seven bacterial indicator strains (4 Gram-positive and 3 Gram-negative) are shown in Table 2. There were variable spectra of inhibition zones of the antibacterial activity of the identified strains against the indicator bacteria which ranged from 8 to 30 mm in diameter. The results indicated antibacterial activity of the CFS of 25 (54.35%), 38 (82.61%), 33 (71.72%), 31 (67.39%), 11 (23.91%), 33 (71.72%), and 38 (82.61%) LAB strains against* E. faecalis*,* E*.* coli*,* Salmonella* spp.,* Shigella sonnei*,* S*.* aureus*, MRSA, and* Listeria monocytogenes*, respectively.* L. casei* MSJ1,* L*.* casei* Dwan5,* L. plantarum* EyLan2, and* E. faecium* Gail-BawZir8 strains showed antibacterial activity against all indicator bacteria at the different recorded spectra of inhibition. 20-30 mm zones of inhibition activity were noticed for* S. thermophilus* BinSlman8 and* S. thermophilus* MaNaL33 against* S*.* aureus* and for* W. confusa* Tarim4 against MRSA. 15.1-20 mm zones of inhibition were recorded for* E. faecium* NSJ2,* S. equinus* Anwr4,* S. equinus* Salam7, and* W. confusa* NooR1 against MRSA and for* E. faecalis* Shbam40 and* L*.* acidophilus* Musallam2 against* Listeria monocytogenes*.

## 4. Discussion

Identified strains (68%) were Gram-positive, catalase-negative, and oxidase- and hemolysis-producing, as well as chain-forming fermentative cocci and rods. These conclusions meet those reached earlier [9, 48] in the study on milk-grown LAB. Our pilot study aimed at the identification of LAB isolated from different types of dairy-based foods obtained from various animals in order to have a complete picture of the LAB found in those products. The results confirmed that the distribution of the isolated LAB is sample-dependent.* Enterococci* were recovered from qeshta, madheer, raw cow milk, and raw goat milk.* Lactobacilli* were isolated from cheese and yogurt made from cow milk.* Streptococci* were abundant in stirred yogurt (Laban) made from camel milk and in frozen camel milk, and* Weissella* were isolated from butter made from cow milk. A phylogenetic tree was successfully generated from the multiple sequence alignment of full- and partial-length 16S rDNA sequences of the 46 strains. We speculate that the LAB of the different genera can be positively discriminated using only the hypervariable region V2. Our data was aligned with that generated by Balcázar et al. [49] who indicated that the sequence containing both the V1 and V2 regions completely discriminated among LAB strains. Chakravorty et al. [50] also indicated that the V1, V3, V5, V6, and V7 regions are conserved, while the V2, V4, V8, and V9 regions are hypervariable in the family.

The antibiotic resistance of probiotic LAB is a controversial subject, as they may be reservoirs of antibiotic genes. The safety of LAB regarding food applications was evaluated by screening for the presence of virulence factors coding genetic determinants and by testing their phenotypic resistance to different antibiotics [51]. It is believed that the bacteria present in the intestinal microflora of food-producing animals may acquire antibiotic resistance. Then, by the exchange of genetic material, the antibiotic-resistant bacteria can transfer the resistance factor to other pathogenic bacteria [33, 52]. To resolve this problem in probiotic studies, it is necessary to certify that a prospective probiotic strain contains no transferable resistance genes. The 15 tested antibiotics were selected for their variety of action mechanisms, revealing different profiles of resistance for the strains similar to the results of Schirru et al. [43]. The different enterococcal strains were sensitive to vancomycin, while Chahad et al. [42] and Haghshenas et al. [46] reported that all tested enterococci were resistant to vancomycin; they claimed that this resistance is an intrinsic property for most LAB. The isolated* L. plantarum *EyLan2 was sensitive to penicillin G, chloramphenicol, gentamicin, and clindamycin in agreement with the results of Haghshenas et al. [46]. However, the presence of antibiotic resistance properties among probiotic bacteria is advantageous as this allows the bacteria to survive in the gastrointestinal tract during antibiotic treatment. In our study, lactobacilli and all other strains were sensitive to aminoglycosides, represented by gentamicin, while very few were resistant to clindamycin. Among streptococci, 100% were sensitive to gentamicin and chloramphenicol, while Federici et al. [11] reported that 16.67% of LAB isolates were resistant to clindamycin and 69.23% and 15.38% of lactobacilli were resistant to gentamicin and clindamycin, respectively, and 75% and 75% of streptococci exhibited resistance to gentamicin and chloramphenicol, respectively.

The absence of cytolysin coding genes is a good characteristic in the food applications of enterococci and other LAB. Cytolysin is a bacterial toxin expressed by some isolates of* E. faecalis* which displays both hemolytic and bactericidal activities [51]. All the 46 studied strains exhibited no *β*-hemolytic activity, which is in agreement with Chahad et al. [42] and Bozoudi et al. [8]. This characteristic confirms that these LAB can be used safely in food applications. The ability of the isolated LAB strains to resist acid and bile is an important probiotic property, since they have to survive the conditions in the stomach and the small intestine [53, 54]. The 46 strains identified in the present study differentially tolerate the acidic and bile conditions; 59% of the isolated LAB were tolerant at a high rate. Washington et al. [3] also indicated that 72% of the tested LAB are tolerant to acidity and bile at a survival rate of 90%, while Messaoudi et al. [53] reported that the* L. salivarius* SMXD51 can tolerate gastrointestinal conditions (pH 3 acidity and 0.5% w/v bile) with a 99% rate of survival. In our study, the behavior of most lactobacilli, especially the strains* L. casei* MSJ1,* L. casei* BgShn3, and* L. Casei* Dwan5, was similar to that observed by Messaoudi et al. [53]. Ladda et al. [55] indicated that* L. paracasei, L. casei, *and* W. confusa* were tolerant to acidity and bile at pH 3.0 and 4% bile, respectively. Bujnakova et al. [33] indicated that* L. salivarius*,* L. agilis, L. reuteri*,* L. murinus*, and* L. amylovorus* were tolerant to acidity and bile at pH 2.5 and 0.3% bile, respectively. The strains of* L. paracasei, L. casei*, and* Lactobacillus rhamnosus* isolated by Reale et al. [2] exhibited high acidity and bile tolerance at pH 3.5 and 1.5% bile, respectively. However, Ahmadova et al. [51] reported that* E. faecium* AQ71 could not grow at pH 3 and 4, while it can grow in the presence of bile concentrations ranging from 0.2% to 0.3%.

As for the antibacterial activity, the largest antimicrobial spectrum in the present study was exerted by CFS of* Lactobacillus casei* MSJ1,* Lactobacillus casei* MasaLam7*, Lactobacillus casei* Dwan5,* Enterococcus faecium* BawZir8,* Lactobacillus paracasei* NMBM1,* Lactobacillus casei* BgShn3, and* Lactobacillus plantarum* EyLan2, as they inhibited all the indicator strains. The indicator strain* E. coli* ATCC 25922 in the present study was sensitive to as many as 38 LAB strains. The LAB isolates studied by Bozoudi et al. [8] almost inhibited the growth of* E. faecalis*,* E. coli* (100%),* S. aureus*, and* L. monocytogenes*. In our study, no inhibition was observed by the isolated* E. faecalis* Shbam40 against* E. faecalis* (ATCC 29212).

Our results indicated that the tested Gram-positive indicator bacteria were more sensitive to the antimicrobial activity of LAB to some extent than the Gram-negative ones, which agrees partially with the results of Ghanbari et al. [56]. Washington et al. [3] indicated no clear relationship between the Gram type of indicator bacteria and their sensitivity to LAB. They also stated that the number of isolated LAB inhibiting the growth of* L. monocytogenes* (ATCC 7644) was greater than the number of those inhibiting* E. faecalis* (ATCC 19433),* E. coli* (ATCC 8739),* Salmonella Typhi* (ATCC 6539),* Shigella flexneri* (ATCC 12022), and* S. aureus* (ATCC 25923). On the other hand, Strompfova and Laukova [24] showed that the growth of all tested Gram-negative indicators was highly inhibited by LAB compared to the inhibition of Gram-positive indicators.

## 5. Conclusions

The raw and fermented milk of animals from Saudi Arabia, especially stirred yogurt (Laban) made from camel milk, was confirmed to be rich in LAB. The most important strains with promising probiotic potential for beneficial applications are* Lactobacillus casei* MSJ1,* Lactobacillus casei* Dwan5,* Lactobacillus plantarum* EyLan2, and* Enterococcus faecium* Gail-BawZir8. We argue that studying the synergistic effects of bacterial combinations might result in the occurrence of a more effective probiotic potential.

## Figures and Tables

**Table 1:** Genotypes of the selected 46 isolated LAB as 16S rDNA gene sequences alignments submitted to the NCBI GeneBank database.

Strain no.	Strain name	GeneBank Acc. No.	Query coverage	Max. ident. %	Isolate source^a^
2	*Enterococcus faecium* Rashad3	KU324893	94%	99%	RCM
3	*Enterococcus durans* Etimad1	KU324894	65%	98%	RCM
4	*Enterococcus faecium* SMBM3	KU324895	98%	99%	RCM
7	*Lactobacillus casei* MSJ1	KU324896	98%	99%	CMCM
8	*Lactobacillus paracasei* NMBM1	KU324897	98%	99%	CMCM
9	*Lactobacillus casei* BgShn3	KU324898	99%	99%	CMCM
10	*Lactobacillus casei* MasaLam7	KU324899	95%	99%	CMCM
11	*Lactobacillus casei* Dwan5	KU324900	96%	97%	CMCM
12	*Lactobacillus plantarum* EyLan2	KU324901	89%	97%	CMCM
19	*Lactobacillus futsaii* EMBM2	KU324902	99%	98%	CCMCM
23	*Weissella confusa* SaEd-7	KU324903	95%	98%	CCMCM
28	*Enterococcus faecalis* Shbam40	KU324904	99%	97%	RGM
29	*Enterococcus faecium* ZiNb3	KU324905	95%	97%	RGM
32	*Enterococcus faecium* Gail-BawZir8	KU324906	93%	97%	SYMCM
34	*Enterococcus faecium* NSJ2	KU324907	99%	96%	SYMCM
35	*Streptococcus thermophilus* BinSlman8	KU324908	99%	99%	SYMCM
36	*Lactococcus lactis* HadRami9	KU324909	98%	98%	SYMCM
37	*Enterococcus faecium* Marwh2	KU324910	97%	99%	QMCM
38	*Enterococcus faecium* Mona3	KU324911	99%	99%	QMCM
39	*Enterococcus faecium* SSJ3	KU324912	98%	97%	QMCM
40	*Enterococcus lactis* Jeddah9	KU324913	97%	98%	QMCM
42	*Streptococcus thermophilus* MaNaL33	KU324914	97%	98%	QMCM
44	*Enterococcus faecium*Adeb3	KU324915	97%	98%	MMGM
45	*Enterococcus faecium* ESJ4	KU324916	99%	96%	MMGM
46	*Enterococcus durans* She7R	KU324917	96%	97%	MMGM
47	*Lactobacillus acidophilus* Hadhramaut4	KU324918	91%	98%	MMGM
50	*Lactobacillus acidophilus* Musallam2	KU324919	99%	97%	YMCM
53	*Enterococcus faecium* BagHom4	KU324920	99%	99%	RCAM
58	*Streptococcus equinus* Omer9	KU324921	95%	97%	RCAM
60	*Streptococcus equinus* Anwr4	KU324922	77%	96%	SYMCAM
61	*Streptococcus equinus* Zaki1	KU324923	98%	98%	SYMCAM
63	*Streptococcus equinus* Salam7	KU324924	87%	96%	SYMCAM
68	*Enterococcus faecium* Ma7Fod	KU324925	98%	98%	SCMCM
73	*Streptococcus equinus* JmaL3	KU324926	96%	96%	FCAM
74	*Streptococcus equinus* Foad7	KU324927	95%	96%	FCAM
75	*Weissella confusa* AhMd8	KU324928	99%	99%	BMCM
76	*Weissella confusa* Tarim4	KU324929	99%	97%	BMCM
77	*Weissella confusa* NooR1	KU324930	78%	95%	BMCM
78	*Weissella confusa* SaYun2	KU324931	98%	98%	BMCM
79	*Weissella confusa* MuKalla5	KU324932	94%	95%	BMCM
80	*Weissella confusa* SYary1	KU324933	98%	97%	BMCM
81	*Weissella confusa* Sho7ir	KU324934	96%	98%	BMCM
82	*Weissella confusa* Farag8	KU324935	97%	97%	BMCM
83	*Weissella confusa* A7Gaf	KU324936	99%	98%	BMCM
92	*Lactococcus garvieae* ZSJ5	KU324937	99%	98%	BMCM
93	*Lactococcus garvieae* Emad4	KU324938	98%	99%	BMCM

^a^RCM: raw cow milk, RGM: raw goat milk, RCAM: raw camel milk, FCAM: frozen camel milk, CMCM: cheese made from cow milk, CCMCM: cooked cheese made from cow milk, SCMCM: salty cheese made from cow milk, SYMCM: stirred yogurt (Laban) made from cow milk, SYMCAM: stirred yogurt (Laban) made from camel milk, YMCM: yogurt made from cow milk, BMCM: butter made from cow milk, QMCM: qeshta (cream) made from cow milk, MMGM: madheer made from goat milk.

**Table 2:** The profile of the selected LAB (n = 46) for main probiotic characteristics and the antibacterial activity spectra of their CFS against seven indicator organisms: A:* Enterococcus faecalis *ATCC 29212, B:* E*.* coli *ATCC 25922, C:* Salmonella* spp., D:* Shigella sonnei *ATCC 25931, E:* Staphylococcus aureus *ATCC25923, F: MRSA ATCC 43330, G:* Listeria monocytogenes *ATCC 13932. −: no inhibition, +: inhibition zone 8.0–10.0 mm, ++: inhibition zone 10.1–15.0 mm, +++: inhibition zone 15.1–20.0 mm, ++++: inhibition zone 20.0–30.0 mm.

Strain no.- code	Sample source	Strain name	Accession no.	Hemolysis	% viability in pH 3 medium^a^	% viability in 0.5% w/v bile^a^	No. of antibiotics sensitive to/ 15	A	B	C	D	E	F	G
2	RCM2	*Enterococcus faecium* Rashad3	KU324893	*γ*-hemolysis	98.1 ± 0.141	98.8 ± 0.141	12	+	++	−	+	−	+	+
3	RCM3	*Enterococcus durans* Etimad1	KU324894	*γ*-hemolysis	99.4 ± 0.141	97.75 ± 0.354	12	+	++	−	+	−	+	+
4	RCM4	*Enterococcus faecium* SMBM3	KU324895	*γ*-hemolysis	99.1 ± 0.283	98.1 ± 0.283	13	+	++	−	+	−	+	+
7	CMCM1	*Lactobacillus casei* MSJ1	KU324896	*γ*-hemolysis	99.45 ± 0.354	99.05 ± 0.212	9	+	++	++	++	+	+	+
8	CMCM2	*Lactobacillus paracasei* NMBM1	KU324897	*γ*-hemolysis	93.55 ± 0.636	98.45 ± 0.212	10	+	+	++	+	−	++	+
9	CMCM3	*Lactobacillus casei* BgShn3	KU324898	*γ*-hemolysis	98.85 ± 0.919	99.25 ± 0.212	9	+	+	+	+	−	+	+
10	CMCM4	*Lactobacillus casei* MasaLam7	KU324899	*γ*-hemolysis	97.5 ± 0.283	97.4 ± 0.424	9	+	++	+	+	−	+	++
11	CMCM5	*Lactobacillus casei* Dwan5	KU324900	*γ*-hemolysis	98.7 ± 0.849	99.3 ± 0.283	9	+	++	+	++	+	+	+
12	CMCM6	*Lactobacillus plantarum* EyLan2	KU324901	*γ*-hemolysis	76.3 ± 0.566	99.0 ± 0.141	11	+	+	+	+	+	+	+
19	CCMCM1	*Lactobacillus futsaii* EMBM2	KU324902	*γ*-hemolysis	98.35 ± 0.778	91.8 ± 1.27	7	++	+	++	+	−	+	++
23	CCMCM5	*Weissella confusa* SaEd-7	KU324903	*α*-hemolysis	99.1 ± 0.141	95.45 ± 0.354	10	−	−	+	−	−	++	+
28	RGM5	*Enterococcus faecalis* Shbam40	KU324904	*γ*-hemolysis	82.2 ± 0.99	67.45 ± 1.626	12	−	++	++	++	−	−	+++
29	RGM6	*Enterococcus faecium* ZiNb3	KU324905	*γ*-hemolysis	99.4 ± 0.141	99.6 ± 0.141	12	+	+	−	+	+	+	++
32	SYMCM3	*Enterococcus faecium* Gail-BawZir8	KU324906	*γ*-hemolysis	99.1 ± 0.283	74.1 ± 2.263	12	++	++	+	+	++	+	+
34	SYMCM5	*Enterococcus faecium* NSJ2	KU324907	*γ*-hemolysis	98.96 ± 0.078	97.6 ± 0.424	11	+	+	+	++	−	+++	++
35	SYMCM6	*Streptococcus thermophilus* BinSlman8	KU324908	*α*-hemolysis	67.35 ± 1.485	95.65 ± 0.636	11	−	+	+	−	++++	−	+
36	SYMCM7	*Lactococcus lactis* HadRami9	KU324909	*γ*-hemolysis	98.95 ± 0.212	65.5 ± 1.98	12	−	+	+	++	−	−	++
37	QMCM1	*Enterococcus faecium* Marwh2	KU324910	*γ*-hemolysis	98.5 ± 0.424	83.65 ± 1.202	12	+	+	−	+	−	+	+
38	QMCM2	*Enterococcus faecium *Mona3	KU324911	*γ*-hemolysis	97.55 ± 0.354	68.15 ± 1.626	11	++	+	+	+	−	+	++
39	QMCM3	*Enterococcus faecium* SSJ3	KU324912	*γ*-hemolysis	98.3 ± 0.283	60.9 ± 1.273	12	++	+	−	+	−	+	+
40	QMCM4	*Enterococcus lactis* Jeddah9	KU324913	*γ*-hemolysis	97.15 ± 0.354	67.15 ± 0.636	11	−	+	+	+	−	+	+
42	QMCM6	*Streptococcus thermophilus* MaNaL33	KU324914	*α*-hemolysis	60.85 ± 1.485	95.1 ± 0.424	11	−	+	+	−	++++	−	+
44	MMGM1	*Enterococcus faecium* Adeb3	KU324915	*γ*-hemolysis	98.5 ± 0.566	97.35 ± 0.636	8	+	++	+	++	−	−	++
45	MMGM2	*Enterococcus faecium* ESJ4	KU324916	*γ*-hemolysis	99.1 ± 0.283	59.8 ± 0.283	12	++	+	−	+	−	+	++
46	MMGM3	*Enterococcus durans* She7R	KU324917	*γ*-hemolysis	95.6 ± 0.424	66.7 ± 1.273	11	++	+	−	+	−	+	++
47	MMGM4	*Lactobacillus acidophilus* Hadhramaut4	KU324918	*γ*-hemolysis	82.65 ± 0.636	90.7 ± 1.131	10	−	++	+	++	−	+	++
50	YMCM3	*Lactobacillus acidophilus* Musallam2	KU324919	*γ*-hemolysis	92.25 ± 0.495	99.45 ± 0.212	11	−	++	+	++	−	+	+++
53	RCAM1	*Enterococcus faecium* BagHom4	KU324920	*γ*-hemolysis	99.5 ± 0.141	98.25 ± 0.495	13	+	++	−	+	−	+	+
58	RCAM6	*Streptococcus equinus* Omer9	KU324921	*γ*-hemolysis	64.55 ± 2.758	97.5 ± 0.283	13	−	+	+	++	−	−	++
60	SYMCAM2	*Streptococcus equinus* Anwr4	KU324922	*γ*-hemolysis	72.85 ± 1.202	97.45 ± 0.495	14	−	+	+	++	−	+++	++
61	SYMCAM3	*Streptococcus equinus* Zaki1	KU324923	*γ*-hemolysis	75.75 ± 1.909	97.1 ± 0.849	14	−	+	+	++	−	−	+
63	SYMCAM5	*Streptococcus equinus* Salam7	KU324924	*γ*-hemolysis	74.45 ± 0.636	98.95 ± 0.212	14	−	+	+	++	−	+++	+
68	SCMCM4	*Enterococcus faecium* Ma7Fod	KU324925	*γ*-hemolysis	98.45 ± 0.636	37.7 ± 0.990	13	+	++	+	++	−	+	+
73	FCAM4	*Streptococcus equinus* JmaL3	KU324926	*γ*-hemolysis	52.85 ± 0.636	95.55 ± 0.636	13	−	+	−	−	+	+	++
74	FCAM5	*Streptococcus equinus* Foad7	KU324927	*γ*-hemolysis	56.75 ± 1.485	95.3 ± 0.566	12	−	+	−	−	+	+	++
75	BMCM1	*Weissella confusa* AhMd8	KU324928	*α*-hemolysis	99.45 ± 0.212	95.3 ± 0.424	10	−	−	+	−	−	−	−
76	BMCM 2	*Weissella confusa* Tarim4	KU324929	*α*-hemolysis	99.15 ± 0.212	95.2 ± 0.424	10	−	−	+	−	−	++++	−
77	BMCM 3	*Weissella confusa* NooR1	KU324930	*α*-hemolysis	99.55 ± 0.071	96.55 ± 0.778	10	++	+	+	−	−	+++	−
78	BMCM4	*Weissella confusa* SaYun2	KU324931	*α*-hemolysis	99.45 ± 0.71	96.35 ± 0.495	10	−	++	++	−	−	−	−
79	BMCM 5	*Weissella confusa* MuKalla5	KU324932	*α*-hemolysis	70.9 ± 0.707	95.95 ± 0.212	11	−	+	−	−	+	++	+
80	BMCM6	*Weissella confusa* SYary1	KU324933	*α*-hemolysis	99.2 ± 0.141	98.2 ± 0.424	11	−	+	−	−	−	−	+
81	BMCM7	*Weissella confusa* Sho7ir	KU324934	*α*-hemolysis	96.9 ± 0.566	94.9 ± 0.849	10	++	−	+	+	+	+	+
82	BMCM8	*Weissella confusa* Farag8	KU324935	*α*-hemolysis	97.3 ± 0.424	95.65 ± 0.636	10	++	−	+	−	−	−	−
83	BMCM 9	*Weissella confusa* A7Gaf	KU324936	*α*-hemolysis	96.75 ± 0.212	95.15 ± 0.636	10	++	−	+	−	−	+	−
92	BMCM 18	*Lactococcus garvieae* ZSJ5	KU324937	*α*-hemolysis	88.5 ± 0.707	98.65 ± 0.354	11	−	−	+	−	−	−	−
93	BMCM19	*Lactococcus garvieae* Emad4	KU324938	*α*-hemolysis	86.0 ± 0.707	99.2 ± 0.141	11	−	−	+	−	−	−	−

^a^Results are presented as the mean value of duplicate trials ± standard deviation (SD).
